# A maternal “mixed, high sugar” dietary pattern is associated with fetal growth

**DOI:** 10.1111/mcn.12912

**Published:** 2019-11-27

**Authors:** Stephanie V. Wrottesley, Alessandra Prioreschi, Sarah H. Kehoe, Kate A. Ward, Shane A. Norris

**Affiliations:** ^1^ SAMRC/Wits Developmental Pathways for Health Research Unit, Department of Paediatrics, Faculty of Health Sciences University of the Witwatersrand Johannesburg South Africa; ^2^ MRC Lifecourse Epidemiology Unit University of Southampton, Southampton General Hospital Southampton UK

**Keywords:** dietary patterns, fetal growth, preconception nutrition, pregnancy, obesity, weight gain

## Abstract

This study examined associations between a maternal “mixed, high sugar” dietary pattern during pregnancy and ultrasound‐determined fetal growth in 495 urban African women and explored whether these associations were independent of maternal baseline body mass index (BMI) and gestational weight gain (GWG). Linear mixed effects modelling (LMM) was used to test the associations between maternal mixed, high sugar dietary pattern score, baseline BMI (kg/m^2^), and GWG (kg/week) and the following fetal growth outcomes: (a) biparietal diameter (cm), (b) head circumference (cm), (c) abdominal circumference (cm), and (d) femur length (cm). In the pooled LMM, a +1 standard deviation (*SD*) increase in the mixed, high sugar dietary pattern score was associated with higher biparietal diameter (0.03 cm/+1 *SD*; *p* = .007), head circumference (0.07 cm/+1 *SD*; *p* = .026), abdominal circumference (0.08 cm/+1 *SD*; *p* = .038), and femur length (0.02 cm/+1 *SD*; *p* = .015). Although these associations were independent of maternal BMI and GWG, higher baseline BMI was independently and positively associated with abdominal circumference (0.03 cm/+1 kg/m^2^; *p* = .011) and femur length (0.01 cm/+1 kg/m^2^; *p* = .007) and 1 kg/week greater GWG was associated with a 0.82 cm increase in abdominal circumference (*p* = .007). In urban African settings, where preconception maternal obesity prevalence is high and processed, high sugar diets are common, improving maternal dietary intake and BMI prior to conception should be prioritised for optimising pregnancy and birth outcomes as well as longer‐term offspring health. In addition, dietary management strategies during pregnancy may be beneficial in facilitating healthy fetal growth.

**Key Messages**

- In transitioning African settings such as South Africa, two thirds of women are overweight or obese prior to conception and urbanisation has been associated with an increase in the consumption of energy dense diets, particularly high in processed foods and added sugar
- This has important implications for maternal and child health in such settings where the risk of developing gestational diabetes and excessive fetal growth and adiposity are high during pregnancy; thus increasing childhood and adolescent obesity risk and perpetuating the cycle of obesity and noncommunicable disease into future generations
- Our study shows that, independent of maternal BMI and gestational weight gain, adherence to a “mixed, high sugar"” dietary pattern ‐ particularly high in table sugar, sweet spreads and high‐sugar breakfast cereals and nut spreads ‐ is associated with higher fetal growth across all fetal growth parameters (i.e. abdominal circumference, femur length, biparietal diameter and head circumference)
- Our findings also indicate a sexual dimorphism in the sensitivity of fetuses to the in utero nutrition environment
- While improving maternal dietary intake and BMI prior to conception should be a focus for optimising pregnancy and birth outcomes, as well as offspring health in the longer term, this data suggests that dietary management strategies during pregnancy may be beneficial in facilitating healthy fetal growth, particularly in obese pregnant women

## 1 INTRODUCTION

In urban African women, maternal dietary patterns during pregnancy have been associated with gestational weight gain (GWG) and with newborn growth and adiposity (Wrottesley, Ong, Pisa, & Norris, 2018; Wrottesley, Pisa, & Norris, 2017), potentially influencing long‐term risk of noncommunicable diseases for mothers and their offspring (Chiavaroli, Derraik, Hofman, & Cutfield, 2016; Godfrey et al., 2017; Holland, Groth, & Kitzman, 2015; Mochizuki, Hariya, Honma, & Goda, 2017). Specifically, in a longitudinal birth cohort study (Soweto First 1000‐Day Study; S1000), we identified three distinct dietary patterns in women during pregnancy using principal component analysis (PCA); namely “western,” “traditional,” and “mixed” (Wrottesley et al., 2017). Dietary transition is well established in highly urbanised settings such as Soweto (South Africa) where the consumption of energy dense and micronutrient poor diets high in refined carbohydrates, processed meats, high fat/sugar convenience foods, and edible oils (i.e., the western and mixed patterns in this case) increasingly surpass that of historically consumed diets high in whole grains, legumes, vegetables, and traditional meats (the traditional pattern; Popkin, 2006; Popkin, Adair, & Ng, 2012; Wrottesley et al., 2017). The S1000 study showed that adherence to the western and mixed dietary patterns was associated with higher maternal weight gain during pregnancy (Wrottesley et al., 2017). In contrast, traditional dietary pattern adherence was associated with lower GWG as well as lower newborn weight‐to‐length ratio and fat mass index (Wrottesley et al., 2018, 2017). These findings have important implications for this setting, where two thirds of women are either overweight or obese prior to conception and higher GWG is not beneficial for mother or baby (Wrottesley et al., 2018, 2017).

Further to the aforementioned findings, analyses from S1000 demonstrated differential associations between dietary patterns during pregnancy and maternal and offspring outcomes according to maternal body mass index (BMI) at baseline (Wrottesley et al., 2018, 2017). In addition, both a higher maternal BMI and greater GWG were positively associated with newborn weight‐to‐length ratio but not with fat mass index (Wrottesley et al., 2018). Differences in the associations between dietary patterns during pregnancy and GWG in normal weight and overweight/obese women have been documented elsewhere (Hillesund, Bere, Haugen, & Øverby, 2014; Tielemans et al., 2015). Additionally, both positive and negative associations between energy dense, processed dietary patterns and birth weight have been shown in previous studies (Coelho, Cunha, Esteves, Lacerda, & Filha, 2015; Colón‐Ramos et al., 2015; Knudsen, Orozova‐Bekkevold, Mikkelsen, Wolff, & Olsen, 2008). This suggests that the complex associations between the in utero nutrition environment and offspring size and adiposity may be highly influenced by maternal nutritional status at baseline, as well as by weight gain during pregnancy.

Although our previous findings are useful in demonstrating associations between maternal nutritional status and dietary patterns and newborn size and adiposity overall, the effects of these exposures on longitudinal fetal growth and on individual fetal growth parameters, that is, femur length, abdominal circumference, biparietal diameter (diameter across the fetuses head, from one parietal bone to the other), and head circumference is of interest (Hinkle, Johns, Albert, Kim, & Grantz, 2015). We have previously demonstrated that gestational diabetes mellitus (GDM) exposure in the S1000 cohort was associated with an increase in fetal biometry—particularly abdominal circumference (Macaulay, Munthali, Dunger, & Norris, 2018). In addition, this effect was specific to male fetuses, suggesting a sexual dimorphism in the vulnerability of fetuses to the in utero nutritional environment (Macaulay et al., 2018).

The aim of this study was therefore to examine the associations between maternal dietary patterns during pregnancy and ultrasound‐determined fetal growth and to determine whether any identified associations were independent of maternal baseline BMI and GWG.

## 2 METHODS

### 2.1 Study setting and participants

This study was nested within a large pregnancy cohort study (S1000), based at the South African Medical Research Council (SAMRC)/Wits Developmental Pathways for Health Research Unit at the Chris Hani Baragwanath Academic Hospital in Soweto, Johannesburg, South Africa between 2013 and 2016. Soweto is a large urban area of Johannesburg where the majority of inhabitants live in low‐income households. Women were recruited from the Antenatal Clinic and Fetal Medicine Unit at Chris Hani Baragwanath Academic Hospital and were eligible for inclusion in the study if they were a black South African (self‐reported ethnicity) who resided in Soweto, or the greater Soweto area, were preferably <14 weeks, but no more than 20 weeks, pregnant with a singleton, and naturally conceived pregnancy, had no known diagnosis of epilepsy or diabetes at the time of recruitment, and were 18 years or older. Data collection for S1000 took place at six time points during pregnancy (<14 weeks, 14–18 weeks, 19–23 weeks, 24–28 weeks, 29–33 weeks, and 34–38 weeks). All women provided written informed consent prior to their inclusion in the study and ethical approval was obtained from the University of the Witwatersrand's Research Ethics Committee (Medical) (M120524). In all, 559 women were recruited into this substudy and had dietary intake assessed at 14–18 weeks.

### 2.2 Data collection

#### 2.2.1 Demographic, health, and socio‐economic variables

Maternal demographic, pregnancy‐related, and socio‐economic variables were collected by trained members of research staff using interviewer‐administered questionnaires at the baseline visit (<14 weeks gestational age). HIV‐status was self‐reported at each pregnancy visit and confirmed using the participant's antenatal clinic card. According to South Africa's national Prevention of Mother‐to‐Child Transmission guidelines (Department of Health, 2014), routine HIV counselling and testing is required during pregnancy, and for any HIV‐positive woman who is not already receiving treatment, antiretroviral treatment (ART) is initiated. All HIV‐positive participants in this study were therefore receiving ART and were stratified according to whether they had been initiated on ART prior to pregnancy (prepregnancy ART) or during the current pregnancy (antenatal ART). Household socio‐economic status was assessed using an asset index which scored each participant according to the number of assets that they possessed out of a possible 11 (electricity, radio, television, refrigerator, mobile phone, personal computer, bicycle, motorcycle/scooter, car, agricultural land, and farm animals). This was based on standard measures used in the Demographic and Health Surveys household questionnaire (available at http://www.measuredhs.com) and has been extensively utilised in this setting (Griffiths, Johnson, Cameron, Pettifor, & Norris, 2013; Kagura et al., 2016).

#### 2.2.2 Maternal anthropometry

A wall‐mounted Stadiometer (Holtain, UK) was used to measure height to the nearest 1 mm during the baseline visit. Weight was measured to the nearest 0.1 kg at each pregnancy visit using a digital scale. Weight at baseline (<14 weeks) was used as a proxy for prepregnancy weight and, together with height, was used to calculate BMI (weight [kg]/height [m^2^]). As there were no underweight women in this sample, BMI was classified according to the following categories: normal weight (18.5–24.9 kg/m^2^), overweight (25–29.9 kg/m^2^), or obese (≥30.0 kg/m^2^). GWG (kg/week) was calculated as ([weight at final pregnancy visit − weight at baseline]/duration of follow‐up). GWG was classified according to the Institute of Medicine's (IOM's) BMI‐specific weight gain ranges (IOM and National Research Council, 2009).

#### 2.2.3 Dietary intake

Habitual dietary intake was assessed at the second pregnancy visit (14–18 weeks) using an interviewer‐administered quantitative food‐frequency questionnaire (QFFQ). This questionnaire is based on a nationally utilised QFFQ which was developed by the SAMRC using data from 11 dietary surveys conducted in rural and urban South Africa (Zingoni, Norris, Griffiths, & Cameron, 2009). It collects data on 214 commonly consumed food items which represent all foods consumed by at least 3% of the population (Nel & Steyn, 2002). The specific QFFQ used has been piloted and modified across age and sex groups in this setting as well as being extensively utilised in previous studies (Wrottesley et al., 2014, 2017; Zingoni et al., 2009). Retrospective data was collected on the frequency and quantity of food and beverage intake during the previous week using food flash cards (high‐quality photographs of food items) and household measures as described elsewhere (Venter, MacIntyre, & Vorster, 2000; Wentzel‐Viljoen, Laubscher, & Kruger, 2011). In addition, these methods were accompanied by the use of two‐dimensional life‐size drawings of foods and utensils and three‐dimensional food models as described and validated by Steyn et al. (in adolescents) and effectively utilised in adult populations in our setting (Steyn et al., 2006; Wrottesley et al., 2014). According to the criteria developed by Dennis et al. for scoring the quality of dietary assessment tools utilised in epidemiological studies (Dennis, Snetselaar, Nothwehr, & Stewart, 2003), the QFFQ used in our study would be classified as a very high‐quality tool—scoring a total of 13 points (high‐quality classified as a score of seven or higher). QFFQ data was captured electronically using REDCap electronic data capture tools hosted at The University of the Witwatersrand (Harris et al., 2009).

#### 2.2.4 Fetal ultrasonography

All women underwent a pregnancy dating scan at baseline. This involved measuring the fetal crown–rump length at <14 weeks (cm), or the biparietal diameter (cm), head circumference (cm), and femur length (cm) in more advanced pregnancies (>14 weeks but <20 weeks). Participants were then invited for follow‐up scans every 5 weeks. Follow‐up visits were at 14–18, 19–23, 24–28, 29–33, and 34–38 weeks gestation. The baseline dating scan was used to calculate gestational age at each subsequent visit and at delivery. The following fetal growth measurements were taken as each follow‐up visit: biparietal diameter, head circumference, abdominal circumference, and femur length. All fetal scans were conducted using a Philips HD‐9 (Philips Ultrasound, Bothell, Washington, USA) ultrasound machine.

### 2.3 Statistical analysis

Data were analysed for 495 pregnant women using STATA 13.0 (StataCorp, College Station, TX, USA). The flow of participants through the substudy to reach the final sample size is depicted in Figure S1.

PCA was used to confirm the previously identified dietary patterns—namely western, traditional, and mixed—as described elsewhere (Wrottesley et al., 2018, 2017). However, due to the comparatively high loadings of high sugar items in the previously labelled mixed pattern, this pattern was reclassified as the “mixed, high sugar” pattern in this study to provide stronger representation of its composition. The more specific classification of the dietary pattern was particularly important for our study given that the “healthier” food items/groups (full‐fat milk and brown and wholemeal bread) would commonly be consumed together with those particularly high in sugar (table sugar, sweet spreads, packaged breakfast cereals, and nut spreads) within the urban‐poor South African setting. PCA was conducted using orthogonal (varimax) rotation on the weekly frequency of consumption of 48 food items/groups. Classification of the QFFQ food items into applicable food groupings was based on those groups described by Crozier et al. (Crozier, Inskip, Godfrey, & Robinson, 2008; Crozier, Robinson, Borland, & Inskip, 2006). The Kaiser–Meyer–Olkin measure of sampling adequacy (0.69) and Bartlett's test of sphericity (*p* < .001) were used to confirm PCA as an appropriate dimension reduction technique for use in this sample. Dietary patterns were retained based on eigenvalues and their visual inflections on a scree plot as well as the percentage of total variance explained. As previously described by Tielemans et al., foods or food groups with factor loadings ≤−0.2 or ≥0.2 reflected strong associations with dietary patterns (Tielemans et al., 2015) and were therefore used to name the patterns. Dietary pattern scores were calculated for each pattern by multiplying factor loadings by the standardised intake frequency of each food/food group and then summing these. Mean factor scores for each dietary pattern were zero, with positive scores representing high and negative scores representing low adherence to the respective pattern (Englund‐Ögge et al., 2014).

Normally distributed continuous variables were presented as mean ± standard deviation (*SD*) and those that were not normally distributed were presented as median (interquartile range; IQR). Categorical variables were described as percentages (%). Differences in fetal growth measurements (biparietal diameter, head circumference, abdominal circumference, and femur length) between male and female fetuses at each visit were analysed using the t‐independent test.

Due to the associations previously demonstrated between the mixed, high sugar dietary pattern and GWG in this population, as well as the associations between this pattern and longitudinal fetal growth parameters during simple regression analyses (data not shown), maternal diet was classified according to adherence to the mixed, high sugar pattern (diet pattern score) in all subsequent analyses. The western and traditional dietary patterns were not found to be associated with any fetal growth outcomes (data not shown).

**Figure 1:**
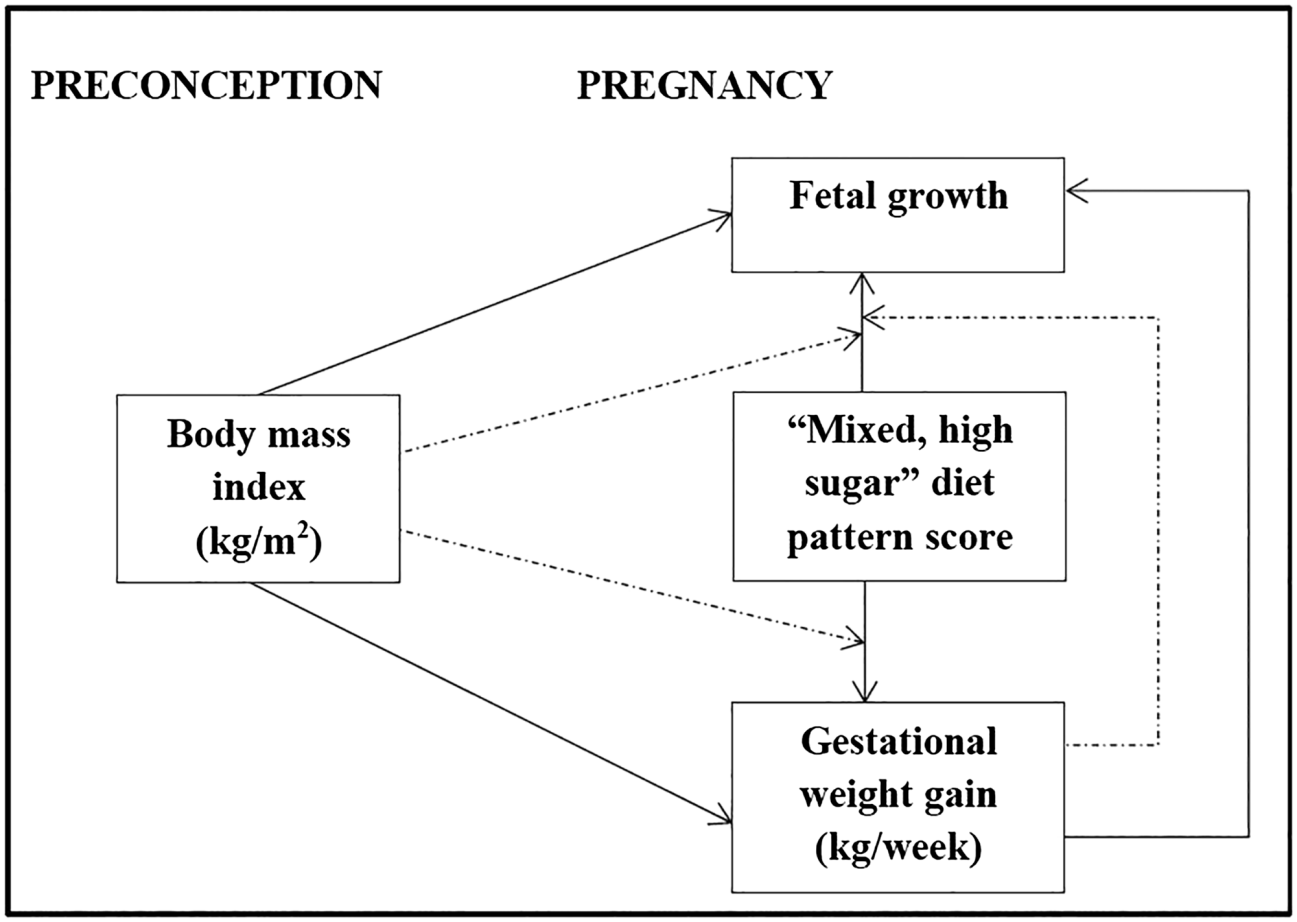
A priori conceptual model for the associations between maternal body mass index (kg/m2), diet (dietary pattern score) and gestational weight gain (kg/week) and longitudinal fetal growth (abdominal circumference, biparietal diameter, femur length, head circumference; cm) assessed via ultrasound at five time points during pregnancy. Solid arrows indicate associations between model variables and dashed arrows indicate where variables may influence an identified association

Based on prior knowledge of the associations between maternal factors and fetal growth from existing studies, as well as previously described associations in this population (Wrottesley et al., 2018, 2017), we proposed an a priori conceptual model for the associations between maternal mixed, high sugar pattern adherence (continuous: diet pattern score), BMI at baseline (continuous: kg/m^2^), GWG (continuous: kg/week), and longitudinal fetal growth during pregnancy (continuous: biparietal diameter, head circumference, abdominal circumference, and femur length; cm; Figure 1). The associations proposed in the conceptual model were then tested for each longitudinal fetal growth outcome using linear mixed effects modelling (LMM; Gibbons, Hedeker, & DuToit, 2010; Hedeker & Gibbons, 2006). All models were run for all participants (*n* = 495) with a random intercept and a random slope as well as with unstructured covariance. Fixed effects for the models per outcome were as follows: Model 1 (M1), fetal sex and mixed, high sugar diet pattern score; and Model 2 (M2), M1 with BMI and GWG. The random effects were the intercept and visit for all models grouped at the individual level. Due to the previous findings by Macaulay et al., a potential interaction with maternal GDM status was also explored. However, the associations between model variables were found to be independent of GDM and therefore this was not included in the final models. Models were run for the pooled dataset and then stratified by fetal sex to explore potential sexual dimorphism in the effects of our exposure variables on fetal growth. A two‐tailed *p* value of <.05 was considered statistically significant. LMM was selected as the modelling approach as it is a widely utilised statistical technique which provides flexibility in the analysis of correlated longitudinal data as well as robust allowance of unbalanced or missing data across study follow‐up (Gibbons et al., 2010; Hedeker & Gibbons, 2006; Pusponegoro, Rachmawati, Notodiputro, & Sartono, 2017).

## 3 RESULTS

### 3.1 Maternal characteristics

Maternal characteristics are presented in Table 1. Median age of pregnant women was 30 years. Thirty‐six percent of women were overweight and 30% were obese at baseline. According to the IoM BMI‐specific guidelines (IOM and National Research Council, 2009), 46% of women gained excessive weight during pregnancy. One third (34%) of women were HIV‐positive and the large majority were nonsmokers (87%). The majority of women were single (62%), living in a medium socio‐economic status level household (83%), and had completed secondary‐level education (73%).

**Table 1:** Characteristics of study participants (*n* = 495)

Participant characteristics	Median (IQR) or %
Demographic and health characteristics	
Maternal age, y	30 (25–34)
Parity	
Para 0	25.3
Para 1	43.8
Para ≥2	30.9
HIV status	
HIV‐negative	65.7
HIV‐positive (antenatal ART)	24.6
HIV‐positive (pre‐pregnancy ART)	9.7
Smokes/chews tobacco	
No	86.9
Yes	13.1
Socioeconomic characteristics	
Maternal education	
Primary	1.6
Secondary	73.1
Tertiary	25.3
Marital status	
Single	61.8
Married/cohabiting	38.2
Household SES	
Low	11.7
Medium	82.6
High	5.7
Anthropometry	
Weight (kg)	68.7 (59.7–78.4)
Height (cm)	158.4 (154.7–162.6)
BMI at baseline, kg/m^2^ (<14 weeks)	27.4 (23.8–30.9)
Normal weight (18.5‐24.9)	33.7
Overweight (25‐29.9)	35.8
Obese (≥30)	30.5
GWG, kg/week	0.35 (0.23–0.46)
Inadequate	29.9
Adequate	24.2
Excessive	45.9

*Note*. IoM GWG ranges (kg/week): inadequate, normal weight <0.35, overweight <0.23, obese <0.17; adequate, normal weight 0.35–0.50, overweight 0.23–0.33, obese 0.17–0.27; excessive, normal weight > 0.50, overweight >0.33, obese >0.27.

Abbreviations: ART, antiretroviral treatment; BMI, body mass index; GWG, gestational weight gain; IQR, interquartile range; SES, socio‐economic status.

### 3.2 Maternal dietary patterns

As described, three dietary patterns were identified in this population, namely western, traditional, and mixed, high sugar. The depicted dietary patterns explained 20.7% of the variation in dietary intake, of which 5.6% was explained by the mixed, high sugar pattern. As previously explained, for the purpose of this study, maternal diet was classified according to adherence to the mixed, high sugar dietary pattern (Table S1). This dietary pattern was relatively heterogeneous but had high loadings for items high in added sugar (table sugar, sweet spreads, packaged breakfast cereals, and nut spreads) as well as some classically healthier foods (brown and wholemeal bread and dairy). The association between the mixed, high sugar dietary pattern and added sugar intake was confirmed in the sample, with those in the highest tertile of adherence to the pattern demonstrating significantly higher added sugar intakes than those in the lowest (T3 vs. T1, mean ± *SD*: 125 ± 106 vs. 76 ± 90 g/d; *p* < .001; data not shown).

### 3.3 Fetal growth

**Figure 2:**
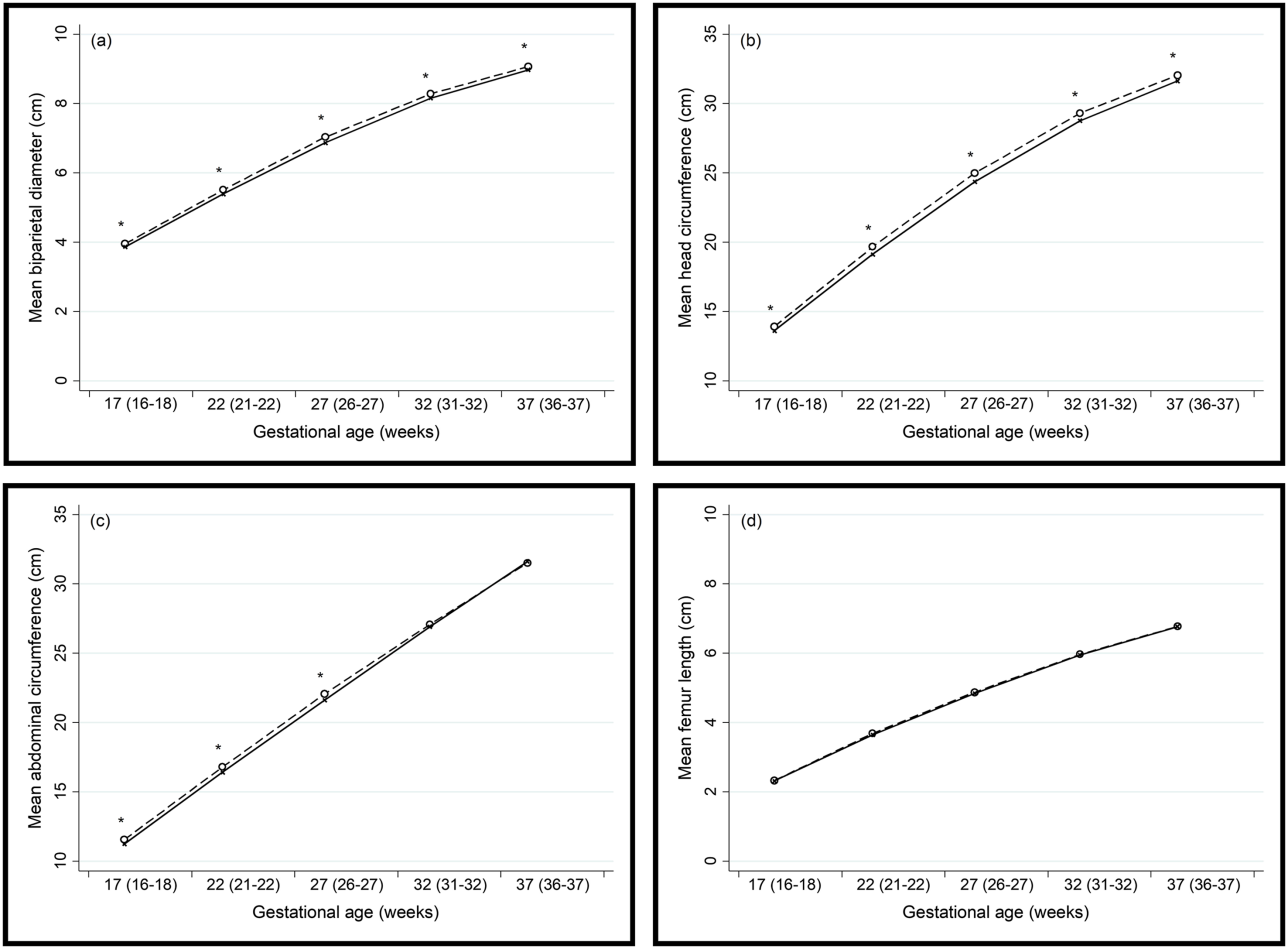
(a) Biparietal diameter (cm), (b) head circumference (cm), (c) abdominal circumference (cm) and (d) femur length (cm) of male (n=256; dashed line) and female (n=239; solid line) fetuses; *Significant difference between males and females (t‐independent test; P<0.05)

Each participant received a median of six (five–six) ultrasound assessments during pregnancy. Ultrasonographic assessment at Visit 1 was used specifically for pregnancy dating (*n* = 495). A total of 2,223 fetal growth scans were therefore performed across the following visits: Visit 2, *n* = 471; Visit 3, *n* = 478; Visit 4, *n* = 469; Visit 5, *n* = 445; and Visit 6, *n* = 360. At each visit, the median gestational weeks were as follows: Visit 2, 17 (16–18) weeks; Visit 3, 22 (21–22) weeks; Visit 4, 27 (26–27) weeks; Visit 5, 32 (31–32) weeks; and Visit 6, 37 (36–37) weeks. Male versus female fetuses had significantly larger mean biparietal diameters and head circumferences at all visits as well as significantly larger abdominal circumferences at Visits 2, 3, and 4 (Figure 2). There were no differences in femur length between male and female fetuses.

Results from the pooled LMM are presented in Table 2. These results show that adherence to the mixed, high sugar dietary pattern was positively associated with all fetal growth outcomes (M1) and that these associations remained when BMI and GWG were included in the models (M2). Specifically, in the final models (M2), greater adherence to the mixed, high sugar dietary pattern was associated with higher biparietal diameter (0.03 cm/+1 *SD* in diet pattern score; *p* = .007), head circumference (0.07 cm/+1 *SD* in diet pattern score; *p* = .026), abdominal circumference (0.08 cm/+1 *SD* in diet pattern score; *p* = .038), and femur length (0.02 cm/+1 *SD* in diet pattern score; *p* = .015). Similarly, when stratified by fetal sex, M2 showed that mixed, high sugar dietary pattern adherence was associated with higher biparietal diameter (0.05 cm/+1 *SD* in diet pattern score; *p* = .001), head circumference (0.15 cm/+1 *SD* in diet pattern score; *p* = .001), abdominal circumference (0.17 cm/+1 *SD* in diet pattern score; *p* = .002), and femur length (0.04 cm/+1 *SD* in diet pattern score; *p* = .002) in male fetuses (Table S2). There were no associations between the mixed, high sugar diet pattern and any fetal growth outcome in female fetuses.

**Table 2:** Results of LMM showing fixed effects on fetal growth outcomes

Fixed effect variables	Biparietal diameter (cm)					
	M1			M2		
	Coefficient	95% CI	*p* value^a^	Coefficient	95% CI	*p* value^a^
Fetal sex						
Male	Ref			Ref		
Female	−0.12	[−0.18, −0.05]	**<0.001**	−0.12	[−0.18, −0.05]	**<0.001**
Maternal diet pattern score						
Mixed, high sugar pattern	0.03	[0.01, 0.05]	**0.005**	0.03	[0.01, 0.05]	**0.007**
BMI, kg/m^2^				0.00	[−0.00, 0.01]	0.405
GWG, kg/week				0.08	[−0.08, 0.25]	0.315
	Femur length (cm)					
	M1			M2		
	Coefficient	95% CI	*p* value^a^	Coefficient	95% CI	*p* value^a^
Fetal sex						
Male	Ref			Ref		
Female	−0.01	[−0.06, 0.05]	0.729	−0.01	[−0.07, 0.04]	0.598
Maternal diet pattern score						
Mixed, high sugar pattern	0.02	[0.01, 0.04]	**0.009**	0.02	[0.00, 0.04]	**0.015**
BMI, kg/m^2^				0.01	[0.00, 0.01]	**0.007**
GWG, kg/week				0.11	[−0.03, 0.24]	0.129
	Abdominal circumference (cm)					
	M1			M2		
	Coefficient	95% CI	*p* value^a^	Coefficient	95% CI	*p* value^a^
Fetal sex						
Male	Ref			Ref		
Female	−0.32	[−0.56, −0.08]	**0.009**	−0.34	[−0.58, −0.10]	**0.005**
Maternal diet pattern score						
Mixed, high sugar pattern	0.09	[0.02, 0.16]	**0.016**	0.08	[0.00, 0.15]	**0.038**
BMI, kg/m^2^				0.03	[0.01, 0.05]	**0.011**
GWG, kg/week				0.82	[0.23, 1.41]	**0.007**
	Head circumference (cm)					
	M1			M2		
	Coefficient	95% CI	*p* value^a^	Coefficient	95% CI	*p* value^a^
Fetal sex						
Male	Ref			Ref		
Female	−0.47	[−0.67, −0.28]	**<0.001**	−0.48	[−0.68, −0.28]	**<0.001**
Maternal diet pattern score						
Mixed, high sugar pattern	0.07	[0.01, 0.13]	**0.021**	0.07	[0.01, 0.13]	**0.026**
BMI, kg/m^2^				0.01	[−0.01, 0.03]	0.185
GWG, kg/week				0.15	[−0.38, 0.65]	0.569

Abbreviations: BMI, body mass index; GWG, gestational weight gain; LMM, linear mixed modelling.

a Significant results are presented in bold (*p* < .05).

Although BMI and GWG did not influence the associations between diet and fetal growth, higher maternal baseline BMI was positively and independently associated with abdominal circumference (0.03 cm per +1 kg/m^2^; *p* = .011) and femur length (0.01 cm per +1 kg/m^2^; *p* = .007) in the pooled models (Table 2). In addition, a 1‐kg/week increase in GWG was associated with a 0.82‐cm increase in abdominal circumference (*p* = .007).

## 4 DISCUSSION

To our knowledge, this is the first study to explore the relationships between maternal dietary patterns and longitudinal fetal growth outcomes in an urban African setting. We found that, independent of baseline maternal BMI and GWG, adherence to a mixed, high sugar dietary pattern during pregnancy was associated with higher biparietal diameter, head circumference, abdominal circumference, and femur length. In addition, we confirmed a sexual dimorphism in the effects of maternal diet on fetal growth—with these associations being specific to male fetuses.

In increasingly overweight and obese populations such as South Africa, over two thirds of women enter pregnancy at risk of developing GDM and/or giving birth to a large fetus (Black, Sacks, Xiang, & Lawrence, 2013). Fetal overgrowth—potentially resulting in delivery of a macrosomic or large‐for‐gestational age infant—substantially increases the risk of maternal and neonatal complications and is also associated with the development of childhood and adolescent overweight and obesity, thereby perpetuating the cycle of obesity and noncommunicable disease risk into future generations (Dabelea & Crume, 2011; Ladfors et al., 2017). Higher measurements of fetal biometry—and a larger abdominal circumference in particular—have been accurately shown to predict fetal macrosomia (Bamberg, Hinkson, & Henrich, 2013). In addition, greater in utero abdominal fat is associated with a higher BMI in childhood, potentially increasing long‐term risk of obesity and metabolic disease in the offspring (Rückinger, Beyerlein, Jacobsen, von Kries, & Vik, 2010). Our study provides novel evidence that a higher sugar diet may increase the risk profile of the fetus by promoting excessive growth across all fetal parameters (i.e., biparietal diameter, head circumference, abdominal circumference, and femur length). In addition, the effects of a 1 *SD* increase in adherence to the mixed, high sugar dietary pattern was consistent across fetal growth measures as well as double that of a +1‐kg/m^2^ higher maternal baseline BMI in the case of abdominal circumference and femur length. Dietary management is therefore critical in at risk women in order to ensure safe pregnancy and delivery for mother and infant as well as to potentially optimise growth, development, and metabolic health of the infant in the longer term.

These results build on previously documented findings showing an association between the mixed, high sugar dietary pattern and higher GWG in women from the S1000 cohort (Wrottesley et al., 2017), suggesting a diet‐associated increase in body size for the mother and now also for the fetus. This dietary pattern was relatively heterogeneous when compared with the western and traditional patterns depicted in the study population and was characterised by high loadings of foods or food groups high in added sugar, which may have been driving these associations. A possible mechanism may be through maternal hyperglycaemia as demonstrated by the findings of Macaulay et al. who showed a positive association between GDM and abdominal circumference in S1000 (Macaulay et al., 2018). In addition, the effects of GDM exposure on fetal growth were not reflected at the time of delivery, with no differences in birth size seen between exposed and unexposed infants. A proposed explanation for this was the identification and management of GDM in the cohort and, therefore, control of the hyperglycaemic state through to delivery. This suggests that dietary management (and reduced intake of added sugar specifically) during pregnancy could have a beneficial effect on growth of the fetus, irrespective of whether the mother develops GDM.

Further to the data from our setting, dietary patterns high in processed foods and/or sugar have been associated with maternal adiposity, GDM risk, and birth size in high‐income and low‐ and middle‐income countries (LMICs; Coelho et al., 2015; Englund‐Ögge et al., 2014; Guilloty et al., 2015; Sedaghat et al., 2017; Tielemans et al., 2015). It has also been suggested that the influence of maternal dietary practices on infant adiposity may persist into childhood. For example, Jen et al. demonstrated an association between sugar‐sweetened beverage intake during pregnancy and higher offspring fat mass index at 6 years of age (Jen et al., 2017). However, inconsistencies in the influence of dietary intakes and patterns on fetal growth and birth outcomes are evident. For example, in some studies from high‐income settings, processed or western dietary patterns have been inversely associated with birth size and typically healthier patterns associated with higher birth weight and reduced risk of a SGA delivery (Colón‐Ramos et al., 2015; Knudsen et al., 2008; Thompson et al., 2010; Timmermans et al., 2012). This may be a result of the complexity between baseline maternal nutritional status, dietary intake during pregnancy, and thus, the maternal metabolic profile; all of which may impact the in utero environment and are associated with offspring outcomes.

Although maternal BMI at baseline and GWG were not shown to influence the associations between diet and fetal growth in our study, these were shown to have independent effects on fetal growth parameters (specifically, femur length and abdominal circumference) and are established predictors of birth size overall (Godfrey et al., 2017; Holland et al., 2015; Wrottesley et al., 2018). Although our findings show that a modified dietary pattern during pregnancy—particularly focused on lower intakes of added (or free) sugar items—may be an effective strategy for reducing the risk of fetal overgrowth, this does not negate the importance of establishing healthy weight profiles prior to conception and effectively managing weight gain throughout pregnancy. In addition, dietary patterns during pregnancy tend to be highly comparable with those preconception (Crozier et al., 2009), suggesting that the dietary patterns depicted in our study are likely to reflect habitual dietary intake in the population. Ensuring healthy dietary patterns and body size preconception is therefore most advantageous for optimising maternal and offspring outcomes.

As previously mentioned, the influence of the maternal mixed, high sugar diet on fetal growth was specific to male fetuses and this has been supported by an effect of GDM exposure (and thus maternal hyperglycaemia) on fetal growth parameters in male, but not female, fetuses (Macaulay et al., 2018). Potential sexual dimorphism in the effects of maternal dietary patterns on fetal growth has been largely overlooked in previous studies, with the majority using only pooled analyses. However, male sex is an established risk factor for adverse pregnancy outcomes, suggesting a greater sensitivity in males to the in utero environment when compared with females (Al‐Qaraghouli & Fang, 2017; Peelen et al., 2016). There are also demonstrated differences in the patterns of fetal and infant growth for males and females as well as sex‐specific differences in the influence of maternal nutritional status and inflammatory state on neonatal fat and lean mass (Broere‐Brown et al., 2016; O'Tierney‐Ginn, Presley, Minium, deMouzon, & Catalano, 2014). This is an important consideration in future studies, as it indicates the potential for sex‐specific programming of early growth and development in response to environmental and biological factors—such as maternal diet and glycaemic profiles—and thus of longer‐term metabolic health risk.

The risks associated with the mixed, high sugar diet are of concern in South Africa, as well as other rapidly transitioning LMICs, which have experienced substantial dietary shifts toward energy dense and micronutrient poor, processed diets high in free sugars. Sugar‐sweetened beverages have been implicated as a key driver of the rising obesity epidemic globally, with the largest increases in beverage consumption occurring in LMICs (Singh et al., 2015). In South Africa, specifically, the growth and implications of the sugar‐sweetened beverage industry have been recognised and taxation on these products was introduced in 2018. However, previous studies have shown that, although sugar‐sweetened beverage intake in South Africa (and wider Southern Africa) has grown, other sources such as table sugar still make substantial contributions to added sugar intakes (Vorster, Kruger, Wentzel‐Viljoen, Kruger, & Margetts, 2014). In our study, which took place prior to the introduction of the sugar tax, the mixed, high sugar dietary pattern was characterised by high sugar food items, specifically table sugar (particularly teaspoons of sugar added to tea) and sweets spreads, as well as packaged breakfast cereals and nut spreads (of which the most commonly purchased and affordable products in South Africa—and in this setting in particular—are high in added sugar). This suggests that, although important, national policies such as sugar‐sweetened beverage taxation alone will not effectively minimise sugar intakes. In low‐income settings such as Soweto, identifying strategies for establishing healthier dietary patterns and weight profiles is challenging due to the substantial barriers to healthy eating in these communities (Ware et al., 2019). These are particularly related to the limited access to, and high cost of, healthy food options and are therefore largely out of the individual's control. Although the need for improved food environments that ensure availability of diverse, low sugar diets is undeniable and needs to be prioritised across South Africa, it is important that young women are empowered to take what steps they can toward healthier lifestyle choices. For example, our study suggests that lower adherence to a dietary pattern driven by added sugar may have a positive effect, with even small daily changes—such as limiting the amount of sugar added to tea—potentially contributing to this. In addition, prioritising meal preparation in the home using unrefined natural ingredients rather than purchasing commercially packaged products such as high sugar breakfast cereals and sugar sweetened beverages, as well as fast food products, in the community should be emphasised and would result in less money spent on “empty calories.”

Our study used an a priori approach to model and test the independent associations between maternal diet and fetal growth, thereby specifying potential covariates to BMI and GWG. Although theoretically sound, this approach does not allow us to understand whether maternal factors—for example, physical activity—may influence these relationships, and this should be explored in future studies. Other limitations of our study include the use of baseline BMI as a proxy for prepregnancy BMI, which may potentially misclassify women who experience weight gain or loss between conception and study recruitment. However, first trimester weight is an established proxy for prepregnancy weight and has been shown to correctly classify 91%–95% of women according to their prepregnancy BMI (Krukowski et al., 2016). In addition, although PCA is an established method for depicting dietary patterns—thereby capturing habitual patterns of intake and providing accessible information for population level recommendations—it is not without flaws. Specifically, the grouping of food items, formatting of the input variables, and naming of the derived patterns is highly subjective (Hu, 2002).

## 5 CONCLUSIONS

Our findings suggest that a maternal dietary pattern high in added sugar during pregnancy may be associated with fetal size. This is of concern in a South African population where preconception maternal obesity prevalence, and therefore risk of greater fetal size and adiposity, as well as physiological programming, is high, and increasingly processed, high sugar diets are common. Although more research is needed to establish the short‐ and longer‐term impact of this association for both mother and child postdelivery, the systematic review by Heslehurst et al. showed a 2.7 times greater odds of a child developing obesity if born to a mother who was obese prior to conception and highlights a transgenerational risk (Heslehurst et al., 2019). Therefore, improving maternal dietary intake and BMI prior to conception should be a key focus for optimising pregnancy and birth outcomes as well as offspring health in the longer term. In addition, dietary management strategies during pregnancy may be beneficial in facilitating healthy fetal growth, particularly in obese pregnant women.

## CONFLICT OF INTEREST

The authors declare that they have no conflicts of interest.

## CONTRIBUTIONS

SVW, AP, and SAN conceptualised and designed the study. SVW was responsible for data management, data analysis, and wrote the original draft. SVW, AP, SHK, KAW, and SAN contributed to interpretation of data and critical review of the manuscript. SAN was the Primary Investigator of the project. All authors gave final approval of the version to be submitted.

## Supporting information

Figure S1. Flow chart of participants within the Soweto First 1000‐Day Study (S 1000) sub‐study
Table S1. Factor loadings of various foods or food groups in the “mixed, high sugar” dietary pattern after principal component analysis (n=495)
Table S2. Results of linear mixed modelling (LMM) showing fixed effects on fetal growth stratified by fetal sex
