# Supplementary material for: A maternal “mixed, high sugar” dietary pattern is associated with fetal growth
Source: Matern Child Nutr. 2019 Nov 27;16(2):e12912. doi: 10.1111/mcn.12912 (PMC7083459; doi:10.1111/mcn.12912)
Supplement: Supplementary file 1 — Figure S1. Flow chart of participants within the Soweto First 1000‐Day Study (S 1000) sub‐study Table S1. Factor loadings of various foods or food groups in the “mixed, high sugar” dietary pattern after principal component analysis (n=495) Table S2. Results of linear mixed modelling (LMM) showing fixed effects on fetal growth stratified by fetal sex [file MCN-16-e12912-s001.docx]

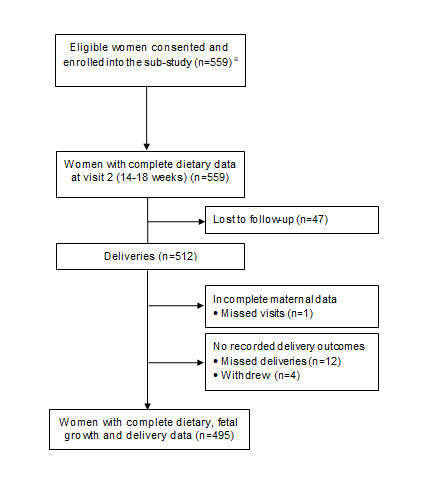


Supplementary Figure 1: Flow chart of participants within the Soweto First 1000-Day Study (S 1000) sub-study ^a^Of eligible women approached at the Antenatal Clinic and Fetal Medicine Unit at Chris Hani Baragwanath Academic Hospital (CHBH), 85% consented to participate. Women who refused to participate were not different in age, BMI, or education, but participants were more likely to be married

Supplementary Table 1: Factor loadings of various foods or food groups in the “mixed, high sugar” dietary pattern after principal component analysis (n=495)

| ***Mixed, high sugar diet pattern*** | |
| --- | --- |
| ***Food or food group*** | ***Factor loading*** |
| **Full-fat milk** | **0.353** |
| **Table sugar (teaspoons)** | **0.350** |
| **Sweet spreads** | **0.345** |
| **Reduced-fat spread** | **0.309** |
| **Brown and wholemeal bread** | **0.280** |
| **Nuts and nut spreads** | **0.271** |
| **Decaffeinated tea and coffee** | **0.221** |
| **Breakfast cereals** | **0.219** |
| Cooked and tinned fruit | 0.187 |
| Tea and coffee | 0.176 |
| Cheese and cottage cheese | 0.153 |
| Fish and seafood | 0.149 |
| Puddings | 0.135 |
| Dried fruit | 0.114 |
| Cakes and biscuits | 0.109 |
| Full-fat spread | 0.087 |
| Yoghurt, buttermilk and maas | 0.086 |
| Fruit juice | 0.079 |
| Quiche and pizza | 0.076 |
| Other vegetables | 0.069 |
| Processed meat | 0.063 |
| Boiled and baked potatoes | 0.053 |
| Salad vegetables | 0.053 |
| Beans and pulses | 0.051 |
| Cream | 0.049 |
| Crackers | 0.042 |
| Root vegetables | 0.042 |
| Other fruit | 0.032 |
| Reduced-fat milk | 0.032 |
| Maize, sorghum and oat porridge | 0.018 |
| Rice and pasta | 0.006 |
| Red meat | -0.001 |
| Cooking fats and salad oils | -0.002 |
| Crisps and popcorn | -0.013 |
| Eggs and egg dishes | -0.014 |
| Sweets and chocolate | -0.023 |
| Miscellaneous (soup powder, condiments, sauces, etc.) | -0.045 |
| Fat cakes and samosas | -0.048 |
| White bread | -0.056 |
| Diet soft drinks | -0.057 |
| Green vegetables | -0.059 |
| Offal and traditional meats | -0.069 |
| Tinned vegetables | -0.070 |
| Roast potatoes and chips | -0.072 |
| Citrus fruit | -0.073 |
| Vegetable dishes | -0.074 |
| Chicken and turkey | -0.095 |
| Soft drinks | -0.185 |
| Explained variance (%) | 5.6 |

Foods or food groups with factor loadings ≥0.2 were classified as characteristic to the dietary pattern and therefore used to describe it (illustrated in bold)

Supplementary Table 2: Results of linear mixed modelling (LMM) showing fixed effects on fetal growth stratified by fetal sex

|  | **BIPARIETAL DIAMETER (cm)** | | | | | | | | | | | |
| --- | --- | --- | --- | --- | --- | --- | --- | --- | --- | --- | --- | --- |
|  | **Male** | | | | | | **Female** | | | | | |
|  | M1 | | | M2 | | | M1 | | | M2 | | |
|  | Coefficient | 95% CI | P-value^a^ | Coefficient | 95% CI | P-value^a^ | Coefficient | 95% CI | P-value^a^ | Coefficient | 95% CI | P-value^a^ |
| Maternal dietary pattern score |  |  |  |  |  |  |  |  |  |  |  |  |
| Mixed, high sugar pattern | 0.05 | 0.02; 0.08 | **0.001** | 0.05 | 0.02; 0.08 | **0.001** | 0.01 | -0.02; 0.04 | 0.445 | 0.01 | -0.02; 0.04 | 0.508 |
| BMI, kg/m^2^ |  |  |  | 0.00 | -0.01; 0.01 | 0.927 |  |  |  | 0.01 | -0.00; 0.01 | 0.218 |
| GWG, kg/week |  |  |  | -0.01 | -0.21; 0.20 | 0.953 |  |  |  | 0.25 | -0.02; 0.52 | 0.068 |
|  | **HEAD CIRCUMFERENCE (cm)** | | | | | | | | | | | |
|  | **Male** | | | | | | **Female** | | | | | |
|  | M1 | | | M2 | | | M1 | | | M2 | | |
|  | Coefficient | 95% CI | P-value^a^ | Coefficient | 95% CI | P-value^a^ | Coefficient | 95% CI | P-value^a^ | Coefficient | 95% CI | P-value^a^ |
| Maternal dietary pattern score |  |  |  |  |  |  |  |  |  |  |  |  |
| Mixed, high sugar pattern | 0.15 | 0.06; 0.24 | **0.001** | 0.15 | 0.06; 0.24 | **0.001** | 0.00 | -0.08; 0.09 | 0.934 | 0.00 | -0.08; 0.08 | 0.999 |
| BMI, kg/m^2^ |  |  |  | 0.01 | -0.01; 0.04 | 0.355 |  |  |  | 0.02 | -0.01; 0.04 | 0.268 |
| GWG, kg/week |  |  |  | -0.11 | -0.74; 0.52 | 0.734 |  |  |  | 0.45 | -0.35; 1.26 | 0.272 |
|  | **ABDOMINAL CIRCUMFERENCE (cm)** | | | | | | | | | | | |
|  | **Male** | | | | | | **Female** | | | | | |
|  | M1 | | | M2 | | | M1 | | | M2 | | |
|  | Coefficient | 95% CI | P-value^a^ | Coefficient | 95% CI | P-value^a^ | Coefficient | 95% CI | P-value^a^ | Coefficient | 95% CI | P-value^a^ |
| Maternal dietary pattern score |  |  |  |  |  |  |  |  |  |  |  |  |
| Mixed, high sugar pattern | 0.18 | 0.07; 0.28 | **0.001** | 0.17 | 0.06; 0.27 | **0.002** | 0.02 | -0.08; 0.12 | 0.718 | 0.01 | -0.09; 0.10 | 0.917 |
| BMI, kg/m^2^ |  |  |  | 0.03 | -0.00; 0.06 | 0.094 |  |  |  | 0.03 | 0.00; 0.06 | **0.040** |
| GWG, kg/week |  |  |  | 0.59 | -0.16; 1.33 | 0.125 |  |  |  | 1.05 | 0.12; 1.99 | **0.027** |
|  | **FEMUR LENGTH (cm)** | | | | | | | | | | | |
|  | **Male** | | | | | | **Female** | | | | | |
|  | M1 | | | M2 | | | M1 | | | M2 | | |
|  | Coefficient | 95% CI | P-value^a^ | Coefficient | 95% CI | P-value^a^ | Coefficient | 95% CI | P-value^a^ | Coefficient | 95% CI | P-value^a^ |
| Maternal dietary pattern score |  |  |  |  |  |  |  |  |  |  |  |  |
| Mixed, high sugar pattern | 0.04 | 0.01; 0.06 | **0.002** | 0.04 | 0.01; 0.06 | **0.002** | 0.01 | -0.01; 0.03 | 0.478 | 0.01 | -0.02; 0.03 | 0.586 |
| BMI, kg/m^2^ |  |  |  | 0.01 | 0.00; 0.02 | **0.018** |  |  |  | 0.01 | -0.00; 0.01 | 0.104 |
| GWG, kg/week |  |  |  | 0.03 | -0.14; 0.21 | 0.720 |  |  |  | 0.20 | -0.02; 0.42 | 0.071 |

Abbreviations: BMI, body mass index; GWG, gestational weight gain ^a^Significant results are presented in bold (p<0.05)
